# A ventral hernia containing appendix; a case report and literature review

**DOI:** 10.1016/j.ijscr.2023.108497

**Published:** 2023-07-12

**Authors:** Majid Samsami, Seyed Pedram Kouchak Hosseini, Alireza Haghbin Toutounchi, Farah Qaderi

**Affiliations:** Department of General Surgery, Imam Hosein Medical and Educational Center, Shahid Beheshti University of Medical Sciences, Tehran, IRAN

**Keywords:** Case report, Abdominal wall hernia, Appendix herniation, Appendicitis, Amyand hernia

## Abstract

**Introduction and importance:**

Abdominal wall hernia is a protrusion of abdominal contents through an acquired or congenital weakness or wall defect. A ventral hernia, including an appendix, is a rare condition. The appendix in the hernial sac is already known as inguinal and femoral hernia and has been named Amyand hernia and De Garengeot hernia, respectively.

**Case presentation:**

We have presented a 74-year-old woman with complaints of point tenderness in the right lower abdomen and para-umbilical and a palpable non-reducible mass over the para-umbilicus with some erythema on the skin, which started two days ago. With the help of imaging, diagnosis of abdominal wall hernia was made, with the appendix as the possible content, as there was evidence of an inflamed appendix lumen incarcerated through the abdominal wall.

**Clinical discussion:**

We have provided a detailed review of recent articles. Our comprehensive discussion includes an exploration of the typical manifestations, the significance of imaging in accurate diagnosis, and the appropriate measures to facilitate optimal surgical preparation. The treatment for ventral hernia typically involves appendectomy and abdominal wall hernia repair, with the specific approach depending on the severity of inflammation.

**Conclusion:**

Although abdominal wall hernia containing appendicitis is extremely rare, its clinical manifestations are hernia and acute appendicitis, the most common diseases in general surgery. Imaging may be helpful in diagnosis. According to our study, diagnostic laparoscopy could be used in case of clinical suspicion of abdominal wall hernia containing an appendix, although more studies are needed.

## Highlights

• Patients with concomitant symptoms of appendicitis and inguinal hernia should have a diagnostic suspicion of ventral hernia.
• Abdominal wall hernia containing appendicitis is an extremely rare phenomenon, its clinical manifestations are hernia and appendicitis.
• diagnostic laparoscopy could be used in case of clinical suspicion of abdominal wall hernia containing appendix.

## 1 Introduction

Abdominal wall hernia is a protrusion of abdominal contents through an acquired or congenital wall defect [1]. When abdominal wall hernias are identified, it is generally recommended to have them evaluated by a surgeon. The specific approach to repair the hernia will depend on factors such as its size and location. Although the incidence is unknown, the National Center for Health Statistics estimates that approximately 5 million Americans have an abdominal wall hernia [1,3]. The factors known to contribute to hernia development include a physiologic change in fascial integrity, proteolysis associated with cigarette smoking, direct mechanical trauma, physical overexertion, abdominal aortic aneurysm, advanced age and familial genetic tendency [1,2,4].

The most common mechanism of hernia formation in the abdominal midline is in the postoperative setting [1]. The contents of the umbilical or para-umbilical hernia are usually omentum, sometimes accompanied by bowel loops; rare contents include metastatic deposits, appendix epiploicae, and a normal or inflamed vermiform appendix. All improbable contents are challenging to diagnose preoperatively, especially in the acute setting. Appendicitis in the hernial sac is known in inguinal and femoral hernias and has been named Amyand hernia and De Garengeot hernias, respectively [5]. Here, we aim to report rare appendicitis within an abdominal wall hernia to highlight the challenges in diagnosis and management. This article has been reported in line with the SCARE 2020 criteria [8].

## 2 Presentation of case

### 2.1 History

A 74-year-old obese woman with a (BMI = 30) presented to our emergency department with respiratory distress and chest pain. During her hospitalization in the cardiology ward, the patient experienced a rapid increase in creatinine levels, necessitating hemodialysis. On the 10th day of hospitalization, a surgical consultation was requested due to the sudden onset of abdominal pain, which the patient had not experienced previously in recent months. Her medical history included controlled hypertension, ischemic heart disease dating back 20 years, and a cholecystectomy performed 15 years ago. There were no notable family history details, and she has not experienced any previous trauma. She is married and has four children. The patient was a non-smoker and did not consume alcohol. She was taking regular oral antihypertensive medications with no reported allergies.

### 2.2 Assessment

During the surgery consult, initial assessment demonstrated that the patient was febrile (38 °C) with blood pressure of 110/70 mmHg and pulse rate of 88/beats per minute. On examination, she was hemodynamically stable, and she had a point tenderness in the right side of the abdomen and para-umbilical and a palpable non-reducible mass over the para-umbilicus with some erythema on the skin, which started two days ago. The pain was mild to moderate in intensity. She had the passage of gas and defecation. She did not have nausea or vomiting. She had a slight loss of appetite. Physical examination was otherwise unremarkable, with no guarding or rebound. There was no evidence of abdominal distension. Laboratory findings included elevated inflammation markers: White Blood Cell count of 24, granulocytes of 87 %, and C-reactive protein level of 7.5 mg/dl. Creatinine was 5 mg/dl, and potassium was 5.9 mmol/l.

**Fig. 1:**
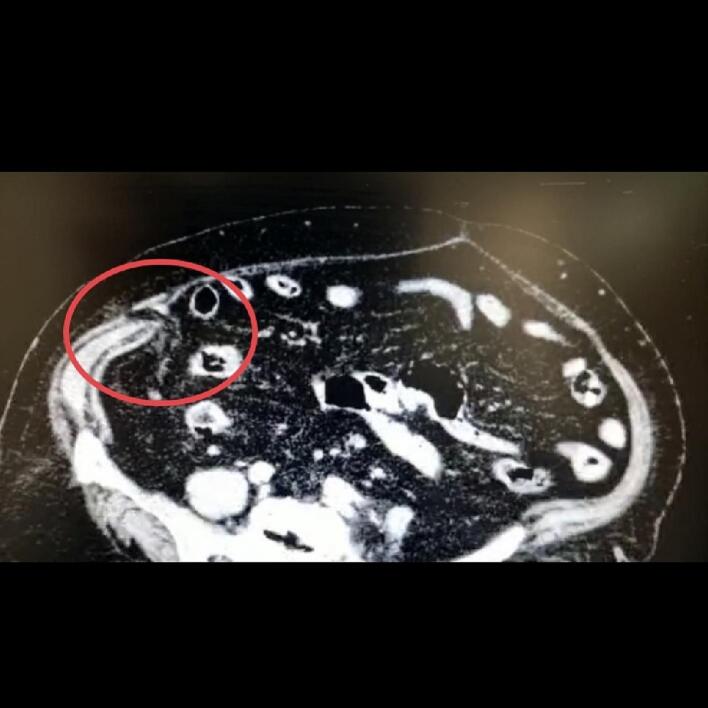
The appendix lumen through the abdominal wall.

Abdominal radiography did not show any signs of free air or air-fluid levels. A CT scan with IV contrast showed a defect of abdominal wall in right quadrant with a 9 mm diameter, including an inflamed appendix and lumen and tended loop with fat stranding within the sac of an abdominal wall hernia, with no associated intra-abdominal free fluid or free air [Fig. 1]. A diagnosis of abdominal wall hernia was made, with the appendix as a possible content (as there were no clinical and radiological features of bowel obstruction).

### 2.3 Operation summary

**Fig. 2:**
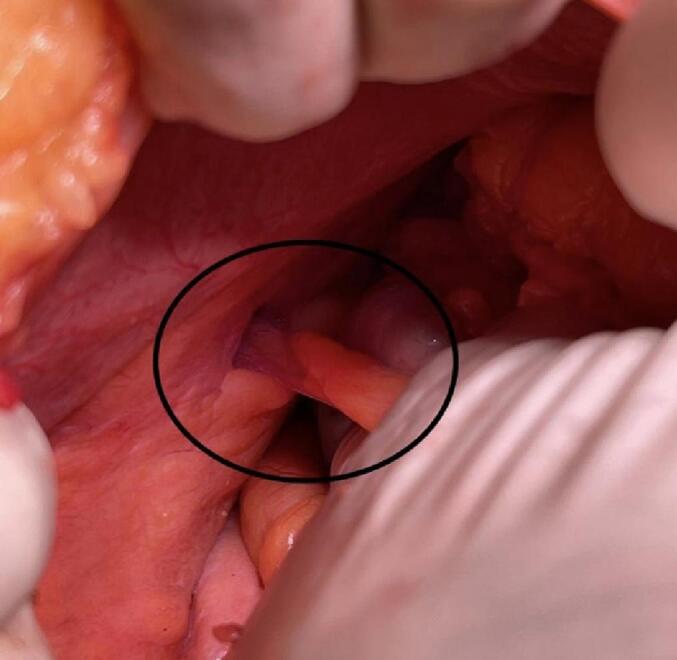
The appendix in facial defect.

The patient underwent dialysis before the operation. Then the appendectomy was performed through a mid-line incision by the attending surgeon. The abdomen was explored with no pathologic point. The sac was opened, and reactive fluid, omentum and an inflamed and edematous appendix were relieved [Fig. 2]. The appendix base was in good condition, so the appendectomy was done, and anatomical repair of the facial defect was performed simultaneously. Postoperatively, the patient underwent antibiotic treatment (ceftriaxone and metronidazole) and routine surgical wound care.

### 2.4 Outcome

The postoperative period was uneventful, and the patient was able to resume per oral intake (PO) a day after the surgery. After 48 h, the patient was discharged and scheduled for follow-up appointments on the tenth day and one month after the procedure, without any complications. Histopathology examination confirmed a diagnosis of acute appendicitis.

## 3 Discussion

Hernias are defined as defects in an aponeurotic layer, resulting in the protrusion of an organ out of a cavity in which it usually resides. This occurs due to increases in intra-abdominal pressure that exceed abdominal wall counter pressure. However, the exact pathogenesis of this entity is not well understood. The factors known to contribute to hernia development include a physiologic change in fascial integrity, proteolysis associated with cigarette smoking, direct mechanical trauma, physical overexertion, and familial genetic tendency [1,6]. Furthermore, a small proportion of these hernias progress to incarceration and even strangulation of the bowel and other viscera, which can be life- threatening [1,4]. The occurrence of the appendix within the abdominal wall is an extremely rare phenomenon. Furthermore, it is rare for patients with abdominal wall hernias with no history of trauma and recent surgery to present with abdominal wall hernia containing appendix and acute appendicitis. In the present case, the patient was found to have neutrophilic leukocytosis indicating an inflammatory process and rise of creatinine. However, such findings are non-specific [7].

There was no evidence of abscess or perforation. Co-morbidities factors such as constipation and risk factors are associated with intermittently increased intra-abdominal pressure, in this case notable. However, the herniated appendix is at a higher risk of developing an infection. It is postulated that the narrow hernial neck causes restricted blood flow, leading to edema and inflammation, and the size of the hernia defect has important implications for recurrence. Surgical experience would suggest that the larger the hernia defect, the more likely the possibility of recurrence. Studies have borne this out, with a threshold of approximately 4 cm representing a 3-fold risk for recurrence [1,2,4].

In the present case, the appendix was found to be inflamed, and a defect of abdominal wall in the right quadrant with 9 mm in diameter as per the CT contrast findings. The presence of appendicitis within the hernia sac has essential surgical implications. For instance, the surgeon needs to perform an appendectomy instead of a simple hernia repair with anatomical fascial defect repair in the case of an abdominal wall hernia with acute appendicitis. Early diagnosis and management of abdominal wall hernia containing appendicitis are crucial, while it may cause infection, recurrence of hernia, edema, abscess, and abstractions of the intestine [1,2]. The widespread use of advanced imaging allowed the preoperative diagnosis of abdominal wall hernia with acute appendicitis. Since inguinal hernia is a clinical diagnosis, patients often undergo surgical repair without needing prior radiological imaging. In the present case, however, the patient was found to have abnormal laboratory markers suggestive of an inflammatory process, which mandated further investigations [4]. We performed a CT scan to rule out any intra-abdominal infection. Although currently, the best and optimal technique for a non- complicated abdominal hernia is the laparoscopic method, in this patient, laparotomy was chosen due to the history of lung problems and COPD [6]. There are controversies about the optimal surgical approach, and the tailored approach in abdominal wall surgery perhaps confirms the greater complexity that is present but does add to the more stringent demands placed on the surgeon [6]. Several factors regarding using mesh to repair the hernia should be considered, including the patient's age, co-morbidities, life expectancy, and the presence of complicated appendicitis.

## 4 Conclusion

While the occurrence of abdominal wall hernias containing appendicitis is exceedingly rare, their clinical presentation includes both hernia and acute appendicitis, which are among the most common conditions encountered in general surgery. Preoperative clinical diagnosis of this phenomenon is challenging, although CT scans and ultrasounds can provide some assistance. Based on our study, diagnostic laparoscopy can be considered in cases where there is a clinical suspicion of an abdominal wall hernia involving the appendix. However, further comprehensive studies are required to determine the optimal preoperative diagnostic approach.

## Consent

Written informed consent was obtained from the patient for publication of this case report and accompanying images. A copy of the written consent is available for review by the Editor-in-Chief of this journal on request.

## Ethical approval

Ethical approval is exempt/waived at our institution (Shahid Beheshti University of Medical Sciences, IRAN) for deidentified case reports.

## Funding

This article did not receive fund.

## Author contribution

Dr. Majid Samsami is the main author and he has designed this report.

Dr. Seyed Pedram Kouchak Hosseini participated in Conceptualization.

Dr. Alireza Haghbin Toutounchi participated in writing and Methodology.

Dr. Farah Qaderi is the writer of this article and corresponding author.

## Guarantor

Dr. Majid Samsami accepts all responsibility of this article.

## Research registration number

Not applicable.

## Declaration of competing interest

All authors declare that they have no conflicts of interest.
